# Knockout of *OsWRKY71* impairs *Bph15*-mediated resistance against brown planthopper in rice

**DOI:** 10.3389/fpls.2023.1260526

**Published:** 2023-11-02

**Authors:** Xiaozun Li, Jian Zhang, Xinxin Shangguan, Jingjing Yin, Lili Zhu, Jie Hu, Bo Du, Wentang Lv

**Affiliations:** ^1^ Shandong Academy of Agricultural Sciences, Jinan, China; ^2^ State Key Laboratory of Hybrid Rice, College of Life Sciences, Wuhan University, Wuhan, China; ^3^ Key Laboratory of Plant Genetics and Molecular Breeding, Zhoukou Normal University, Zhoukou, China; ^4^ State Key Laboratory of Ecological Pest Control for Fujian and Taiwan Crops, Fujian Agriculture and Forestry University, Fuzhou, China

**Keywords:** rice, brown planthopper, *Bph15*, *OsWRKY71*, defense mechanism, transcriptome

## Abstract

The *Bph15* gene, known for its ability to confer resistance to the brown planthopper (BPH; *Nilaparvata lugens* Stål), has been extensively employed in rice breeding. However, the molecular mechanism by which *Bph15* provides resistance against BPH in rice remains poorly understood. In this study, we reported that the transcription factor *OsWRKY71* was highly responsive to BPH infestation and exhibited early-induced expression in *Bph15*-NIL (near-isogenic line) plants, and OsWRKY71 was localized in the nucleus of rice protoplasts. The knockout of *OsWRKY71* in the *Bph15*-NIL background by CRISPR-Cas9 technology resulted in an impaired *Bph15*-mediated resistance against BPH. Transcriptome analysis revealed that the transcript profiles responsive to BPH differed between the *wrky71* mutant and *Bph15*-NIL, and the knockout of *OsWRKY71* altered the expression of defense genes. Subsequent quantitative RT-PCR analysis identified three genes, namely sesquiterpene synthase *OsSTPS2*, EXO70 family gene *OsEXO70J1*, and disease resistance gene *RGA2*, which might participate in BPH resistance conferred by *OsWRKY71* in *Bph15*-NIL plants. Our investigation demonstrated the pivotal involvement of *OsWRKY71* in *Bph15*-mediated resistance and provided new insights into the rice defense mechanisms against BPH.

## Introduction

The brown planthopper (BPH; *Nilaparvata lugens* Stål) is a major rice (*Oryza sativa*) pest and a representative piercing-sucking insect. The most effective strategy for managing BPH is the cultivation of insect-resistant rice varieties (Zhang, 2007). Research has identified and characterized a total of 34 major BPH resistance genes, with fifteen of them having been isolated (Du et al., 2020; Chen et al., 2022). Among these genes, *Bph14* encodes a nucleotide-binding and leucine-rich repeat (NLR) protein, which interacts with OsWRKY46 and OsWRKY72, ultimately leading to the activation of downstream defensive genes in rice (Du et al., 2009; Hu et al., 2017). A recent discovery has revealed that Bph14 can directly bind BPH effector BISP, leading to the activation of effector-triggered immunity (ETI) (Guo et al., 2023). *Bph6* encodes a unique leucine-rich repeat (LRR) protein that is specifically localized within the exocyst complex and exhibits interactions with OsEXO70E1 and OsEXO70H3. This interaction promotes protein secretion and strengthens the plant cell wall (Guo et al., 2018; Wu et al., 2022). *Bph30* encodes a novel protein comprising two leucine-rich domains, which is expressed in sclerenchyma cells and enhances the deposition of hemicellulose (Shi et al., 2021). *Bph3*, which was discovered in Rathu Heenati (RH), encodes three lectin receptor kinases (*OsLecRK1*-*OsLecRK3*) that are located on the plasma membrane and function as receptors in plant immunity (Liu et al., 2014). The *Bph15* locus, derived from *Oryza officinalis*, is a compound locus consisting of two genes. One of these genes is identical to *Bph3* (Cheng et al., 2013a; Xiao et al., 2016), while the other major genetic locus has been localized to the adjacent 580-kb recombination cold-spot region (Lv et al., 2014). The resistance to BPH is mediated through a molecular mechanism that shares similarities with defense mechanisms against pathogens (Cheng et al., 2013b; Du et al., 2020; Chen et al., 2022). *Bph15* and *Bph3* potentially function as receptors for recognizing herbivore-associated molecular patterns (HAMPs), thereby initiating pattern-triggered immunity (PTI). However, the precise molecular mechanisms underlying the functions of *Bph15* and *Bph3* remain poorly elucidated.

In the realm of plants, WRKY transcription factors (TFs) represent a substantial assemblage of TFs, characterized by a discernible affinity for the W-box sequence situated within the promoter regions of their respective target genes (Rushton et al., 2010). The influence of WRKY transcription factors on the immune responses of plants is both significant and extensive (Eulgem and Somssich, 2007). In rice, numerous WRKY TFs have been identified as positive regulators of plant pathogen resistance, exemplified by the enhanced resistance against diverse pathogens observed in *OsWRKY13*, *OsWRKY30*, *OsWRKY45*, and *OsWRKY89* (Qiu et al., 2007; Shimono et al., 2007; Wang et al., 2007; Peng et al., 2012). However, the role of *OsWRKY45* is of significant interest due to its documented importance in providing the rice R protein Pb1 against fungal pathogens, albeit with a negative impact on resistance against the BPH (Inoue et al., 2013; Huangfu et al., 2016). Additionally, another WRKY transcription factor, *OsWRKY53*, has been discovered to act as a positive regulator of plant pathogen and BPH resistance. Moreover, it has been identified as a negative feedback regulator of *MPK3* in response to the herbivorous striped stem borer (SSB) (Chujo et al., 2007; Hu et al., 2015; Hu et al., 2016). The activation of *OsWRKY70* in response to BPH attack has been demonstrated to result in a reduction in gibberellic acid (GA) production in rice, leading to an increased susceptibility to BPH (Li et al., 2015). In general, WRKY TFs play a significant role in regulating plant immune response and can have both positive and negative effects on defense against various pests and pathogens in rice.

Among the members of the OsWRKY IIa subfamily, *OsWRKY28*, *OsWRKY62*, *OsWRKY71*, and *OsWRKY76* are encompassed (Peng et al., 2010). Among these genes, *OsWRKY28* and *OsWRKY76* function as negative regulators of basal defense responses against blast fungus (Chujo et al., 2013; Yokotani et al., 2013). *OsWRKY62* functions as a suppressor of basal and *Xa21*-mediated defense against the bacterial pathogen (Peng et al., 2008). In the absence of *Pi9*, *OsWRKY62* assumes a beneficial role in regulating blast resistance; however, in the presence of *Pi9*, it assumes an adverse role (Shi et al., 2023). The involvement of *OsWRKY71*, which is activated by biotic elicitors and pathogen invasion, plays a significant role in the defense response of rice. Overexpression of *OsWRKY71* enhances rice’s resistance to *Xoo*13751, resulting in the constitutive expression of two marker genes, *OsNPR1* and *OsPR1b* (Liu et al., 2007). Furthermore, *OsWRKY71* can interact with the promoter region of the blast resistance gene *GF14b*, thereby influencing its expression (Liu et al., 2016). In addition, *OsWRKY71* also functions as a transcriptional inhibitor (Chujo et al., 2008). Previous studies showed that the overexpression of *OsWRKY71* leads to an increase in H_2_O_2_ accumulation, resulting in enhanced resistance against the small brown planthopper (*Laodelphax striatellus*, SBPH) in rice (Ji et al., 2021). However, its role in regulating resistance against the BPH remains unclear.

Our previous research indicated that the expression of *OsWRKY71* is up-regulated in the *Bph15* introgression line following BPH feeding (Lv et al., 2014). This study demonstrated that *OsWRKY71* plays a critical role in the *Bph15*-mediated resistance against BPH. The knockout of *OsWRKY71* resulted in an impaired BPH resistance in *Bph15*-NIL. Transcriptome sequencing analysis demonstrated distinct transcript profiles between the *OsWRKY71* knockout plants and *Bph15*-NIL plants, suggesting that *OsWRKY71* is involved in regulating the expression of plant defense genes. Further investigation using qRT-PCR identified three genes, namely sesquiterpene synthase *OsSTPS2*, EXO70 family gene *OsEXO70J1*, and disease resistance gene *RGA2*, which may participate in the BPH resistance conferred by *OsWRKY71* in *Bph15*-NIL plants.

## Materials and methods

### Plant materials and insects

As previously stated by Lv et al. (2014), the near-isogenic line *Bph15*-NIL (XF07-151) was found to possess the resistance gene *Bph15* in the Taichung Native 1 (TN1) background. This particular line, *Bph15*-NIL, was employed as a control in the study due to its demonstrated resistance. In contrast, the varieties TN1 and Nipponbare (NIP) were selected as susceptible rice controls. The BPH insects, which were reared on the TN1 variety, were utilized for plant feeding during the research conducted at Wuhan University in China.

### Development of mutants and overexpression transgenic plants

The *wrky71* mutants were generated using CRISPR-Cas9 technology (Ma et al., 2015). The target sequence of *WRKY71* was designed and used to generate sgRNA expression cassettes driven by the OsU6a promoter. Using an Agrobacterium-mediated technique, the resulting structure was transformed into *Bph15*-NIL plants. The confirmation of T_0_ transgenic plants was achieved through genomic targeting by directly sequencing PCR products. The identification of homozygous mutant plants was conducted using PARMS (Penta-primer Amplification Refractory Mutation System) genotyping technology, and these plants were subsequently cultivated for bioassay purposes (Lv et al., 2019).

To construct plasmids for the overexpression of *OsWRKY71*, a 1047 bp coding sequence (CDS) fragment of *OsWRKY71* (accession no. AY676927) was amplified from *Bph15*-NIL (**Supplementary Table S1**). The PCR product was subsequently introduced into the binary vector pCXUN (accession no. FJ905215). Using an *Agrobacterium*-mediated technique, the resulting structure was transformed into Nipponbare and *Bph15*-NIL plants. The T_2_ homozygous plants were grown for the following bioassay.

### BPH resistance evaluation

A progeny test was conducted to obtain the rice resistance scores. The seedling stage of rice varieties was evaluated for resistance against BPH, with each cultivar or line being replicated a minimum of three times. Eighteen seeds were collected from individual plants and planted in plastic cups, or fifty seeds were randomly planted in plastic boxes. At the three-leaf stage, eight nymphs of BPH (second- or third-instar) were introduced to infest the seedlings. The plant condition was assessed and assigned a resistance score ranging from 0 to 9, with 9 indicating the highest susceptibility and 0 indicating the highest resistance level, as explained in previous research (Huang et al., 2001).

### BPH weight gain assays

The assessment of BPH insect performance on rice encompassed the utilization of assays to quantify BPH weight gain and weight gain rate. BPH was introduced to seedlings at the four-leaf stage after the planting of each line’s seeds in a plastic cup with a diameter of 10 cm. Newly emerged female BPH insects were weighed and enclosed in parafilm sachets, which were then affixed to the leaf sheath of a seedling at the four-leaf stage. The BPH instars were allowed to feed on the rice crops for 48 hours. After this feeding period, each insect was removed from the sachet and reweighed. The BPH weight gain was determined by measuring the difference between the initial and final weights of the BPH insect. Furthermore, we computed the weight gain rate, which is determined by dividing the weight gained by the insect’s initial weight before feeding. For each cultivar or line, a minimum of three replicates, each containing 10 insects, were utilized.

### RNA-seq and data analysis

Stems of three-leaf stage *Bph15*-NIL and *wrky71* mutant *wrky71-5-9* were harvested for 0 (uninfested control), 3, and 24 HAI (hours after BPH infestation). All treatments were terminated at the same time, as previously described (Lv et al., 2014). The *Bph15*-NIL samples were designated as R0, R3, and R24, while the *wrky71-5-9* samples were labeled as S0, S3, and S24. The RNA-seq analysis was conducted using an Illumina Novaseq™ 6000 (LC-Bio Technology CO., Ltd.).

Using the Illumina paired-end RNA-seq technique, transcriptome sequencing was performed, resulting in a total of 305 million paired-end reads with a length of 2×150 bp. Following the application of Cutadapt, the reads underwent filtration and purification, resulting in 42 Gbp of reads with high quality. Subsequently, the cleaned reads were aligned to the rice reference genome using HISAT2. The estimation of transcript expression levels was conducted using the StringTie and Ballgown software, and the expression abundance was determined by calculating the FPKM values. The DESeq2 software was employed for performing differential expression analysis between distinct groups. Genes displaying a p-value below 0.05 and an absolute fold change exceeding 2 were categorized as differentially expressed genes (DGEs). Furthermore, the DGEs underwent subsequent analysis to ascertain the enrichment of functions associated with Gene Ontology (GO) and KEGG pathways. The bioinformatics analysis was carried out utilizing the OmicStudio tools accessible at https://www.omicstudio.cn/tool.

### Quantitative RT-PCR

The stems of three-leaf stage *Bph15*-NIL, *wrky71-5-9*, and TN1 were collected after BPH infestation for 0, 3, 6, 12, 24, and 48 HAI. All treatments were terminated simultaneously. The tissue expression profile samples included different rice tissues: stem and leaf of 10 days seedling; stem, sheath, and leaf at the heading stage; stem, sheath, and leaf at 7 days after pollination. The TRIzol reagent (Invitrogen) was employed for the extraction of total RNA. Subsequently, the PrimeScript RT Reagent Kit (Takara) was utilized to perform reverse transcription of 1 μg of total RNA from each sample into cDNA. The qRT-PCR analysis was performed using the QuantStudio™ 5 (Applied Biosystems) and gene-specific primers (**Supplementary Table S1**) in conjunction with the TB Green (TaKaRa) in the PCR system. The internal control TBP (LOC_Os03g45410) was utilized to standardize the results, and the gene expression levels were determined using the 2^-ΔΔCt^ method. Three biological replicates were conducted for each gene in the qRT-PCR analysis.

### Subcellular localization analysis

The coding sequences of *OsWRKY71* were amplified through PCR utilizing the primers OsWRKY71-YFP (**Supplementary Table S1**). Subsequently, the sequences were cloned downstream of a ubiquitin promoter, in frame with YFP, within the vector pCAMBIA1300. The resulting construct was designated as OsWRKY71-YFP. To serve as a nucleus marker, a bZIP63-CFP expressing construct was developed and cloned into the pGWB17 vector. The expression constructs were simultaneously introduced into rice protoplasts. The fluorescence was detected using a confocal laser-scanning microscope (FV1000, Olympus).

### Statistical analyses

The data were subjected to statistical analysis using a one-way ANOVA in either Microsoft Excel or SPSS software (version 20).

## Results

### *OsWRKY71* is early induced by BPH in *Bph15*-NIL

**Figure 1:**
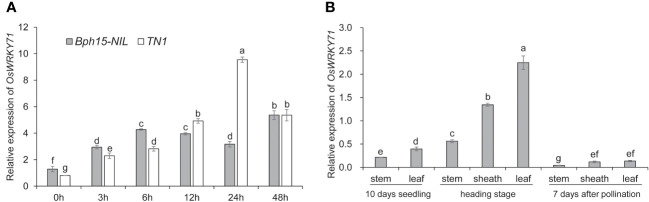
The expression profile of *OsWRKY71*. **(A)** Time-dependent expression of *OsWRKY71* in the resistant plant *Bph15*-NIL and susceptible plant TN1 after BPH infestation. **(B)** Tissue expression profile of *OsWRKY71* in various organs. Data represent the means ± SD. The average is determined by calculating the mean of three biological repeats. Significant differences (p < 0.05) are indicated by varying letters above the bars, as determined by a one-way ANOVA.

In our previous study, it was observed that the expression of *OsWRKY71* increased in response to BPH feeding in the *Bph15* introgression line (Lv et al., 2014). To further investigate the role of *OsWRKY71* in the response to BPH, we conducted an analysis of its expression in both the *Bph15*-NIL and TN1 rice plants at various time points following BPH infestation (0, 3, 6, 12, 24, and 48 HAI). The expression level of *OsWRKY71* in the *Bph15*-NIL was significantly higher than in TN1 at 0, 3, and 6 HAI, but this expression pattern was reversed at 12 and 24 HAI (**Figure 1A**). This suggests that the expression profile of *OsWRKY71* in *Bph15*-NIL differs from that of TN1. Specifically, *OsWRKY71* is early induced by BPH in *Bph15*-NIL. Tissue expression analysis demonstrated that the expression level of *OsWRKY71* is higher in the leaf and sheath tissues at the heading stage compared to other tissues (**Figure 1B**). Therefore, *OsWRKY71* may play a role in *Bph15*-mediated BPH resistance.

### OsWRKY71 is localized in the nucleus

**Figure 2:**
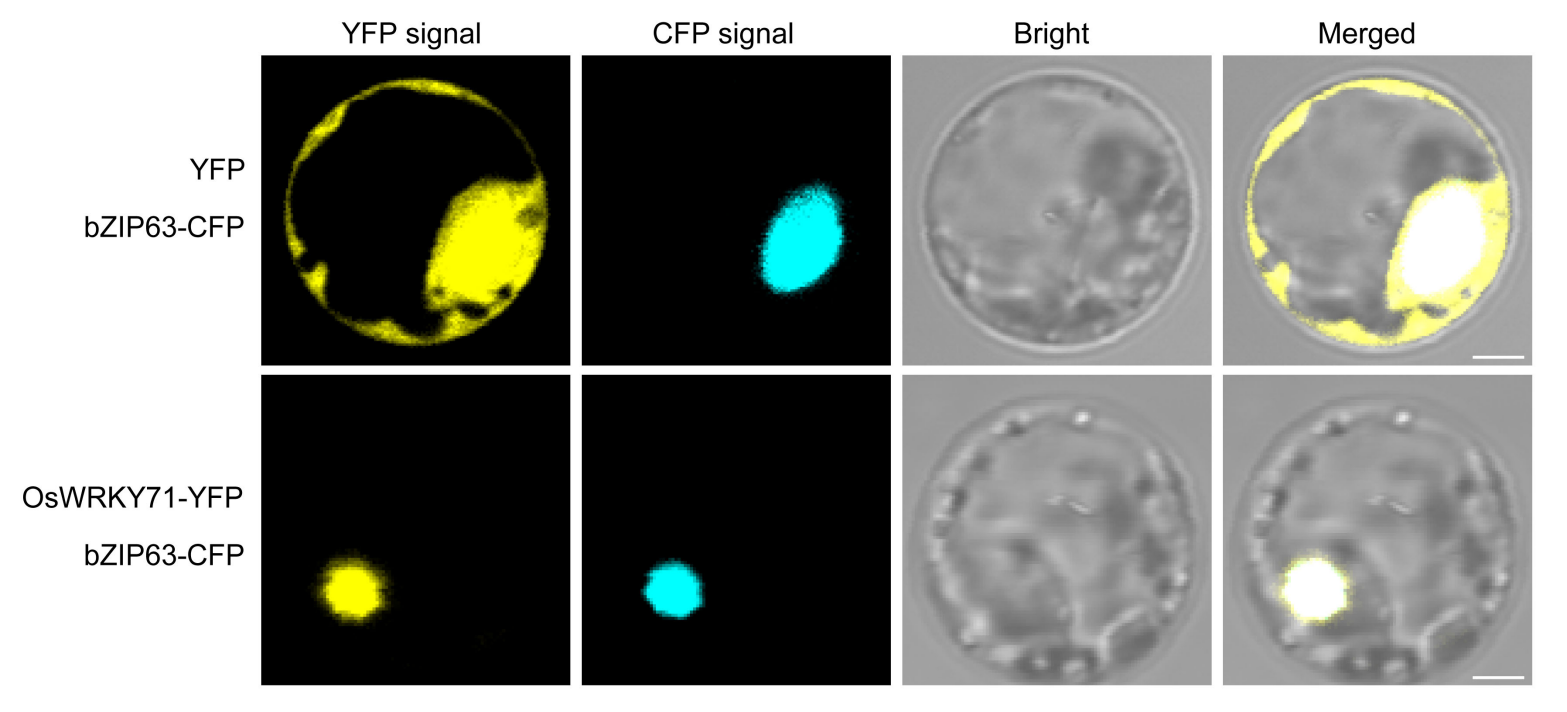
OsWRKY71 subcellular localization. The OsWRKY71-YFP and nucleus marker bZIP63-CFP were co-expressed in rice protoplasts. Scale bars, 2.5 μ m.

Inconsistent subcellular localization of OsWRKY71 has been reported in previous studies (Liu et al., 2007; Kim et al., 2016; Ji et al., 2021). The present study reveals a significant colocalization between OsWRKY71 and the nucleus marker bZIP63 in rice protoplasts, thereby providing substantial evidence supporting the nuclear localization of OsWRKY71 (**Figure 2**). This localization pattern is consistent with the well-established role of transcription factors, which primarily function within the nucleus to regulate gene expression.

### Knockout of *OsWRKY71* impairs the *Bph15*-mediated resistance against BPH

**Figure 3:**
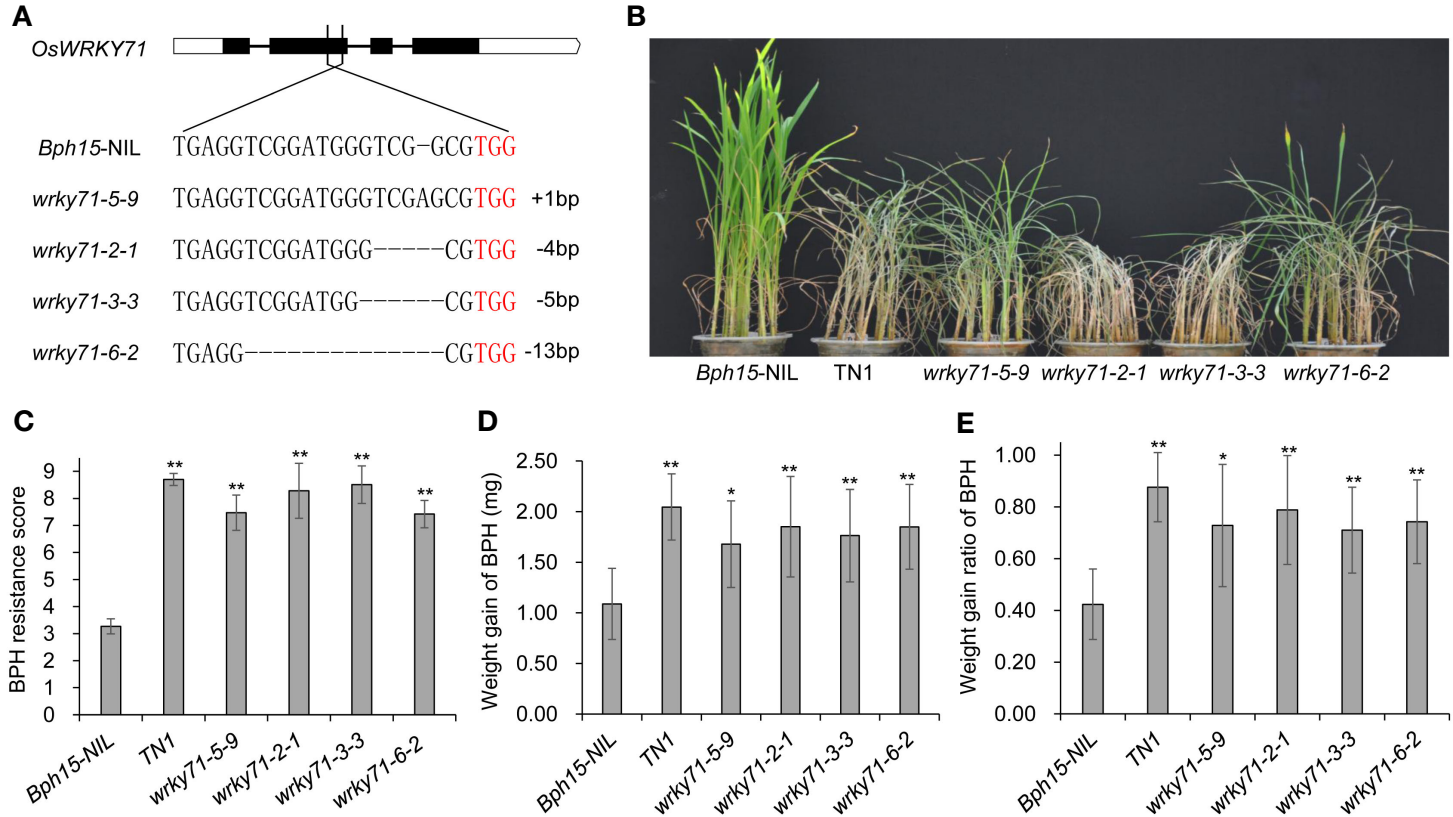
BPH resistance test of the *OsWRKY71* knockout plants. **(A)** Four representative homozygous *wrky71* mutants in the *Bph15*-NIL background. TGG (red) is the PAM sequence. **(B)** BPH resistance evaluation of *wrky71* mutants. **(C)** BPH average resistance scores of *wrky71* mutants. Data represent the means ± SD from three separate experiments, each consisting of 15 seedlings per rice line. Significant differences in comparison with *Bph15*-NIL are indicated by asterisks above the bars (**p < 0.01 by one-way ANOVA). **(D, E)** BPH weight gain and weight gain rate assays of *wrky71* mutants. Data represent the means ± SD from three separate experiments, each consisting of 10 BPH insects per replicate. Significant differences in comparison with *Bph15*-NIL are indicated by asterisks above the bars (**p < 0.01, *p < 0.05 by one-way ANOVA).

To further understand the role of *OsWRKY71* in *Bph15*-mediated resistance, we used CRISPR-Cas9 technology to knockout *OsWRKY71* in the *Bph15-NIL* background. Consequently, we achieved the creation of four distinct homozygous mutant plants, denoted as *wrky71-5-9*, *wrky71-2-1*, *wrky71-3-3*, and *wrky71-6-2*, within the T_2_ generation. Subsequent DNA sequencing analysis unveiled that each mutant exhibited distinct genetic alterations within the targeted region of the *OsWRKY71* gene. It was observed that *wrky71-5-9* had an insertion of A, *wrky71-2-1* had a deletion of 4 base pairs, *wrky71-3-3* had a deletion of 5 base pairs, and *wrky71-6-2* had a deletion of 13 base pairs (**Figure 3A**). The BPH resistance scores of the *wrky71* mutants were evaluated, revealing that *wrky71-5-9*, *wrky71-2-1*, *wrky71-3-3*, and *wrky71-6-2* plants were susceptible. Conversely, the *Bph15*-NIL plants, which possessed intact *OsWRKY71*, exhibited resistance and survived the BPH attack (**Figure 3B**). The mean resistance score for *Bph15*-NIL plants was determined to be 3.27, indicating their resistance to BPH. Conversely, the *wrky71* mutants exhibited significantly higher resistance scores, ranging from 7.42 to 8.51, thereby suggesting their susceptibility to BPH (**Figure 3C**). Additionally, BPH insects that consumed the *wrky71* mutants displayed a greater increase in weight compared to those that consumed *Bph15*-NIL plants (**Figures 3D, E**). These findings provide evidence that the knockout of *OsWRKY71* impairs the resistance of *Bph15*-NIL plants to BPH, thereby emphasizing the crucial role of *OsWRKY71* in *Bph15*-mediated resistance.

We also generated overexpression lines of *OsWRKY71* in both the Nipponbare and *Bph15*-NIL backgrounds. However, the overexpression of *OsWRKY71* in both backgrounds did not result in significant differences in BPH resistance compared to the control plants. Specifically, the *Bph15*-NIL-OE plants, which overexpressed *OsWRKY71* in the *Bph15*-NIL background, exhibited BPH resistance scores similar to those of the *Bph15*-NIL control plants. This suggests that the overexpression of *OsWRKY71* did not enhance the resistance provided by *Bph15* (**Supplementary Figure S1A**). In a similar vein, the NIP-OE plants, characterized by the overexpression of *OsWRKY71* in the Nipponbare background, exhibited susceptibility to BPH and were killed by BPH at the same time as the susceptible Nipponbare plants (**Supplementary Figure S1B**). These observations imply that increasing the expression of the *OsWRKY71* gene does not exert any discernible influence on BPH resistance, irrespective of the presence or absence of *Bph15*. Consequently, it can be deduced that *OsWRKY71* does not function as a positive regulator of basal resistance in the absence of *Bph15*.

### The transcript profiles that respond to BPH are different in the *wrky71* mutant and *Bph15*-NIL

**Figure 4:**
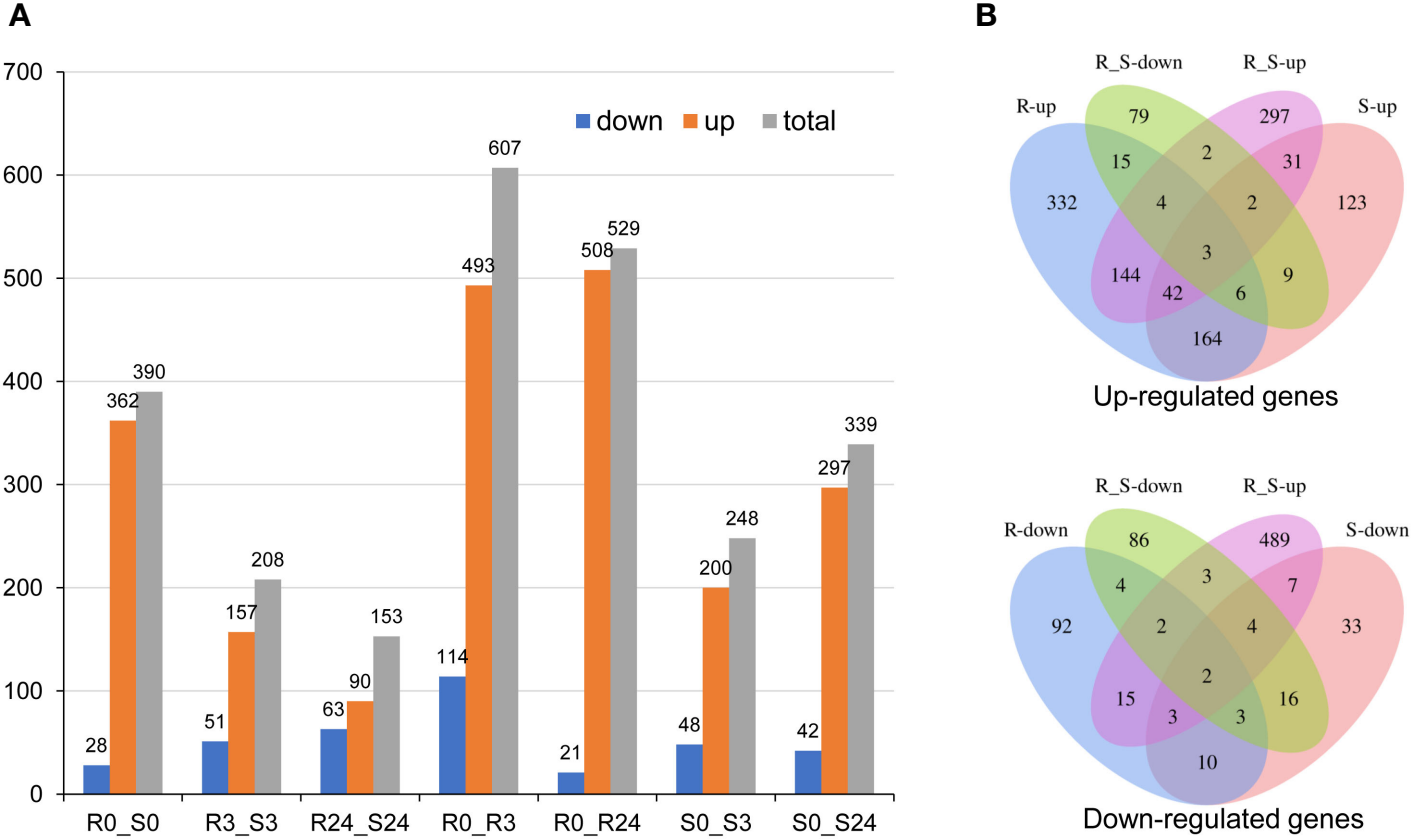
DEGs of two rice varieties (*Bph15*-NIL and *wrky71-5-9*). **(A)** The DEGs in all comparisons. **(B)** Venn diagrams of the up-regulated and down-regulated DEGs.

To investigate the potential mechanism underlying the impact of the *wrky71* mutant on *Bph15*-mediated resistance to BPH, we conducted an analysis of the transcriptomes of *wrky71-5-9* and *Bph15*-NIL using RNA-seq. The RNA-seq analysis identified a total of 1387 differentially expressed genes (DEGs) among seven comparisons: R0_S0, R3_S3, R24_S24, R0_R3, R0_R24, S0_S3, and S0_S24 (**Supplementary Table S2**). Significant disparities in gene expression were observed between the *wrky71* mutant and *Bph15*-NIL. Specifically, in the comparisons of R0_S0, R3_S3, and R24_S24, the *wrky71* mutant displayed significant alterations in 390, 208, and 153 genes, respectively, when compared to *Bph15*-NIL. Moreover, the *wrky71* mutant displayed 248 and 339 DEGs in the S0_S3 and S0_S24 comparisons, respectively. Conversely, in *Bph15*-NIL, there were 607 and 529 DEGs in the R0_R3 and R0_R24 comparisons, respectively. Notably, both the R and S comparisons exhibited a higher abundance of up-regulated genes compared to down-regulated genes (**Figure 4A**). Subsequent analysis revealed significant disparities in the majority of the up-regulated and down-regulated genes between the *wrky71* mutant and *Bph15*-NIL (**Figure 4B**). Additionally, hierarchical cluster analysis of 1387 DEGs further confirmed substantial distinctions between the R and S comparisons (**Supplementary Figure S2**).

**Figure 5:**
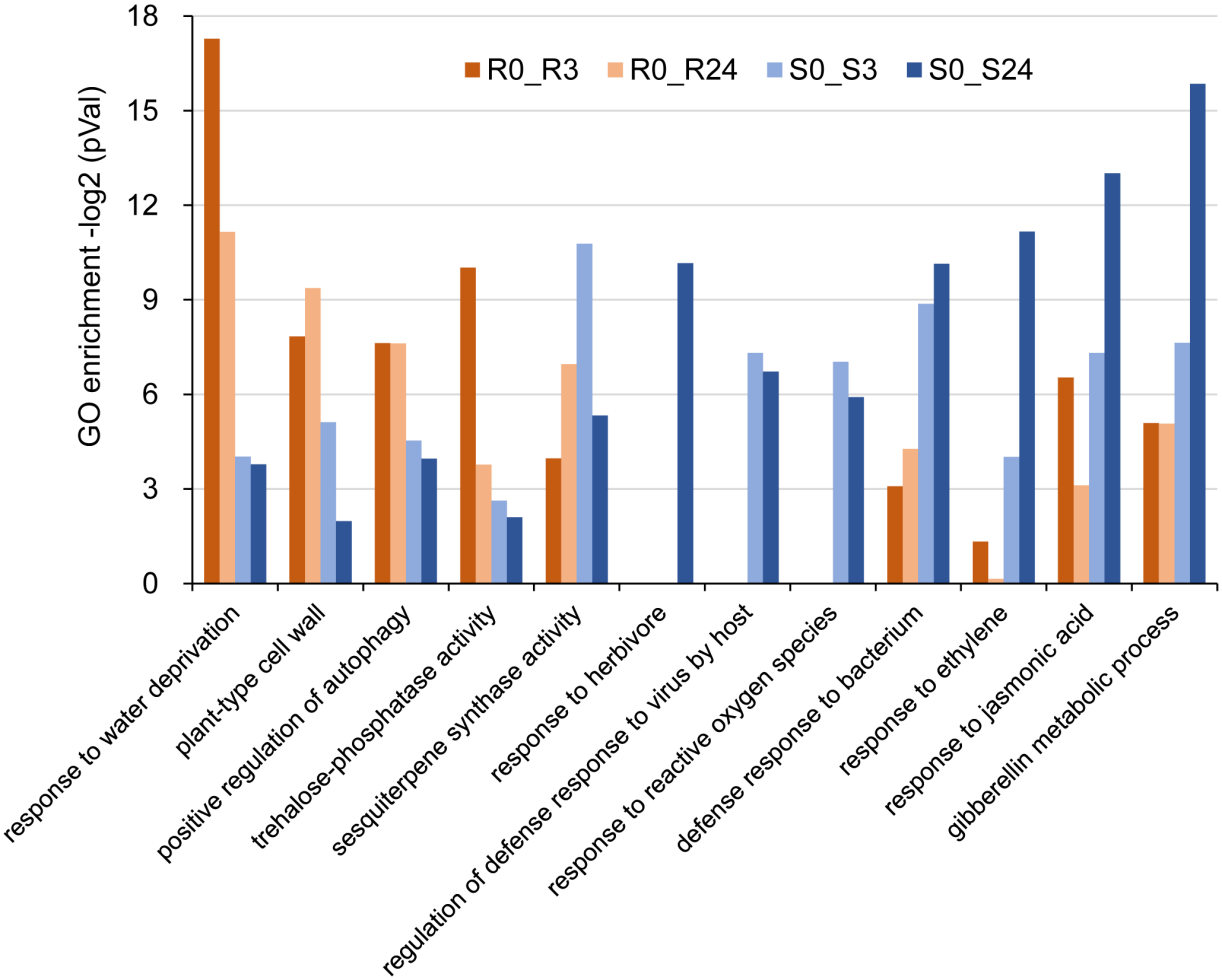
Representative GO terms of up-regulated DEGs in R0_R3, R0_R24, S0_S3, and S0_S24 comparisons. vertical coordinate: -log_2_(P-value). -log_2_(P-value) > 6.64 = p < 0.01, -log_2_(P-value) > 4.32 = p < 0.05.

The enriched Gene Ontology (GO) terms identified in this study suggest that the response to BPH feeding in both the *wrky71* mutant and *Bph15*-NIL rice involves multiple key biological processes. Specifically, the common GO terms observed in the up-regulated DEGs (**Supplementary Figure S3**; **Table S3_1**) in the S0_S3, S0_S24, R0_R3, and R0_R24 comparisons include wounding response, chitin response, fungus defense, and salicylic acid (SA) response. These shared GO terms indicate that both the *wrky71* mutant and *Bph15*-NIL rice employ similar defense mechanisms in response to BPH feeding. Significant disparities in enriched GO terms were observed when comparing gene expression between the *wrky71* mutant and *Bph15*-NIL rice. Specifically, the R0_R3 and R0_R24 comparisons revealed enriched GO terms related to water deprivation response, cell wall, autophagy, and trehalose phosphatase. Conversely, the S0_S3 and S0_S24 comparisons exhibited specifically enriched GO terms associated with ethylene (ET) response, jasmonic acid (JA) response, gibberellin metabolic, bacterium defense, virus defense, and herbivore response (**Figure 5**; **Supplementary Tables S3_2-S3_5**). The enriched GO term “sesquiterpene biosynthetic” in both the R0_R24 and S0_S3 comparisons suggests that this biological process may play a crucial role in conferring resistance to BPH in both the *wrky71* mutant and *Bph15*-NIL rice (**Figure 5**). These results emphasize the potential contribution of diverse biological processes and signaling pathways in the resistance mechanisms of the *wrky71* mutant and *Bph15*-NIL rice, providing valuable insights for future investigations on BPH resistance in rice.

The KEGG pathway enrichment analysis demonstrated significant enrichment in multiple pathways within the transcript profiles of both the *wrky71* mutant and *Bph15*-NIL plants upon exposure to BPH infestation. Notably, these pathways encompassed plant-pathogen interaction, plant hormone signal, autophagy, et al. (**Supplementary Figure S4**; **Table S4_1**). Intriguingly, the R0_R3 and R0_R24 comparisons exhibited a higher number of genes involved in these pathways compared to the S0_S3 and S0_S24 comparisons (**Supplementary Figure S5**; **Tables S4_2-S4_5**). This observation implies the existence of discrete regulatory mechanisms functioning in the two genotypes in response to BPH infestation. Overall, these findings provide insights into the molecular mechanisms of BPH resistance in rice and underscore the importance of *OsWRKY71* in governing gene expression during BPH infestation.

### Knockout of *OsWRKY71* alters the expression of plant defense genes

**Figure 6:**
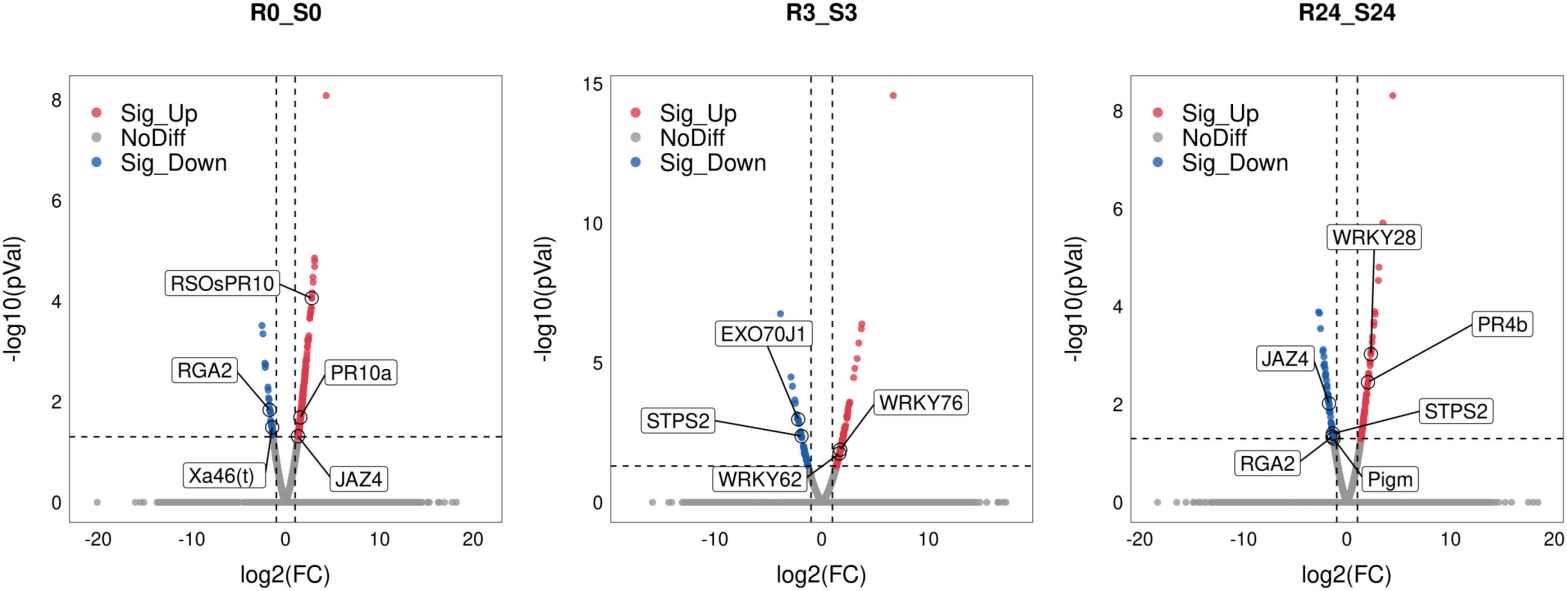
Volcano plots of R0_S0, R3_S3, and R24_S24 comparisons. The representative DEGs were marked as boxes.

**Figure 7:**
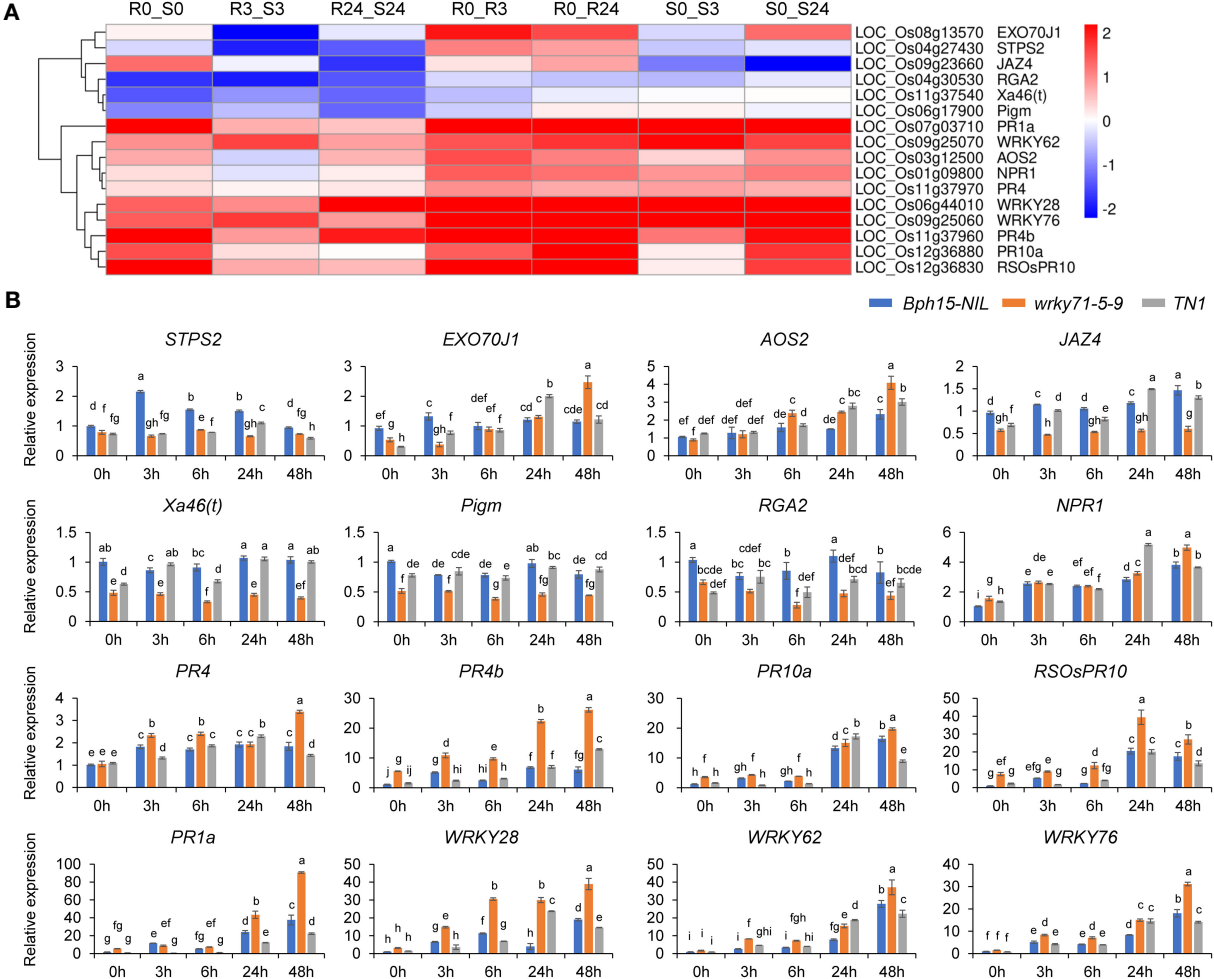
Heat map and qRT-PCR analysis of representative DGEs. **(A)** Heat map of representative DGEs studied in this work. **(B)** qRT-PCR analysis of representative DGEs. Data represent the means ± SD. Significant differences (p < 0.05) are indicated by varying letters above the bars, as determined by a one-way ANOVA.

The study revealed a significant down-regulation of the sesquiterpene synthase gene *OsSTPS2* in the R3_S3 and R24_S24 comparisons (**Figure 6**). Subsequent analysis using qRT-PCR demonstrated a notable decrease in expression levels of *OsSTPS2* in both the *wrky71* mutant and TN1 compared to *Bph15*-NIL at all five time points (**Figure 7**). Terpenes, a class of secondary metabolites, possess diverse functions, including defense against herbivores. Previous research has demonstrated the differential expression of *OsSTPS2* in rice cultivars RH (carrying *Bph3*) and KD (sensitive to BPH), and its involvement in BPH antixenosis resistance (Kamolsukyunyong et al., 2013; Kamolsukyeunyong et al., 2021). This suggests that *OsSTPS2* may participate in BPH resistance conferred by *OsWRKY71* in *Bph15*-NIL plants.

The excretion of proteins associated with resistance and the plant’s resistance to BPH are influenced by the trafficking pathway that relies on the presence of EXO70. Specifically, the functions of *OsEXO70E1* and *OsEXO70H3* are involved in *Bph6*-mediated BPH resistance (Guo et al., 2018; Wu et al., 2022). Our investigation led to the discovery of the *OsEXO70J1* gene (LOC_Os08g13570), a member of the EXO70 family, which exhibited significant up-regulation in the R0_R3 and R0_R24 comparisons, but down-regulation in the R3_S3 comparison (**Figure 6**; **Supplementary Figure S6**). Additionally, the results of qRT-PCR analysis showed that the expression levels of *OsEXO70J1* were reduced in both the *wrky71* mutant and TN1 cultivars compared to *Bph15*-NIL at the onset of BPH infestation (**Figure 7**). This suggests that *OsEXO70J1* may participate in BPH resistance conferred by *OsWRKY71* in *Bph15*-NIL plants.

Interestingly, the down-regulation of three R genes, namely *Xa46(t)* (LOC_Os11g37540), *Pigm* (LOC_Os06g17900), and *RGA2* (LOC_Os04g30530), was observed in the R_S comparisons (**Figure 6**). It was found that BPH feeding did not induce the up-regulation of these genes in any of the rice lines. However, the expression levels of these genes were significantly reduced in the *wrky71* mutant compared to *Bph15*-NIL. Additionally, the expression level of *RGA2* was also decreased in TN1 when compared to *Bph15*-NIL plants (**Figure 7**). These findings suggest that *OsWRKY71* may be involved in controlling the expression of these three R genes and contribute to disease resistance in rice. It is also suggested that the R gene *RGA2* may have a dual function in both disease and BPH resistance.

The influence of the SA and JA pathways on plant responses to insects is of significant importance. However, our previous study suggested that the SA signaling pathway may not be triggered in *Bph15*-mediated resistance (Lv et al., 2014). In this current investigation, we focused on the JA pathway and found that the expression levels of the *AOS2* gene, which is involved in JA synthesis, were increased in both the *wrky71* mutant and TN1 compared to *Bph15*-NIL. Furthermore, the transcript levels of the JA repressor *JAZ4* were lower in the *wrky71* mutant compared to *Bph15*-NIL and TN1 (**Figure 7**). The results indicate that *OsWRKY71* may inhibit the JA pathway in *Bph15*-NIL plants. Hence, the *Bph15*-mediated BPH resistance may not be associated with the SA and JA signaling pathways.

The present study observed an increase in the expression of *NPR1* and five PR genes (*PR1a*, *PR4*, *PR4b*, *PR10a*, and *RSOsPR10*) in all three rice lines. However, the expression levels of these genes were higher in the *wrky71* mutant compared to *Bph15*-NIL plants (**Figure 7**). This suggests that these genes may be involved in a common defense mechanism in both resistant and susceptible rice varieties. These results are consistent with our previous study (Lv et al., 2014). It was observed that the transcript levels of *OsWRKY28*, *OsWRKY62*, and *OsWRKY76*, members of the OsWRKY IIa subfamily, exhibited a significant increase in all three rice lines. However, in the *wrky71* mutant, their expression levels were even higher compared to *Bph15*-NIL plants (**Figure 7**). This suggests that in the absence of *Bph15*, these genes may act as promoters of BPH resistance, but in the presence of *Bph15*, they could exert an inhibitory effect. These findings are consistent with the previous studies conducted by Chujo et al. (2013) and Shi et al. (2023).

## Discussion

BPH resistance genes *Bph15* and *Bph3* may act as pattern recognition receptors (PRRs) in the activation of PTI for plant immunity (Du et al., 2020; Chen et al., 2022). The present study has revealed the significant contribution of *OsWRKY71* in the *Bph15*-mediated resistance against BPH. The knockout of *OsWRKY71* has been observed to impair the resistance to BPH in *Bph15*-NIL plants, as depicted in **Figure 3**. These findings underscore the role of *OsWRKY71* in the underlying molecular mechanisms of *Bph15*-mediated resistance against BPH. In our previous studies, we successfully cloned the first BPH resistance gene *Bph14*, and observed the interaction between the BPH14 protein and OsWRKY46 and OsWRKY72 (Du et al., 2009; Hu et al., 2017). Further research about how *OsWRKY71* is induced by *BPH15* and BPH infestation will enhance our comprehension of the signaling pathways implicated in rice defense against BPH.

The study by Liu et al. (2007) and Ji et al. (2021) reported that *OsWRKY71* enhances resistance to bacterial blight and SBPH in the Nipponbare variety of rice. However, our study found that overexpression of *OsWRKY71* did not increase resistance to BPH in both the Nipponbare and *Bph15*-NIL backgrounds. This suggests that *OsWRKY71* may have varying effects on resistance to BPH and other pathogens or piercing-sucking insects. Similar contrasting roles have been observed for another gene, *OsWRKY45*, in rice pathogens and BPH resistance (Huangfu et al., 2016).

Interestingly, the *OsWRKY71* gene displayed an early induced expression pattern in *Bph15*-NIL, while its level of gene expression was comparatively lower than that of the TN1 at 12 and 24 HAI (**Figure 1A**). However, little is known about the underlying mechanism, and more research needs to be done. The induced expression pattern in TN1 suggests that *OsWRKY71* may potentially contribute to a basal defense in susceptible rice varieties. However, it is important to note that the NIP-OE plants, which overexpress *OsWRKY71*, exhibited susceptibility to BPH infestation (**Supplementary Figure S1B**). This indicates that *OsWRKY71* does not function as a promoter of basal resistance in the absence of *Bph15*. Previous studies have demonstrated that *OsWRKY62* can play opposite roles depending on the existence or nonexistence of the blast-resistance gene *Pi9* (Shi et al., 2023). Similarly, it is plausible that *OsWRKY71*, akin to *OsWRKY62*, possesses a dual capacity in governing the basal resistance against BPH in rice. Nevertheless, further research is required to ascertain whether *OsWRKY71* acts as a negative modulator of BPH basal defense.

Previous research has provided evidence for the involvement of the plant sesquiterpene synthase *OsSTPS2* in an antixenosis mechanism of BPH resistance mediated by *Bph3* (RH variety) (Kamolsukyunyong et al., 2013; Kamolsukyeunyong et al., 2021). In this study, we observed that the expression of *OsSTPS2* was suppressed in the *wrky71* mutant within the *Bph15*-NIL genetic background (**Figure 7**). This finding suggests that *OsSTPS2* may play a part in the antixenosis process of BPH resistance regulated by *Bph15*, and its regulation is mediated by the *Bph15*-*OsWRKY71* pathway. Our future investigations aim to identify the specific plant volatile compounds released by the *wrky71* mutant and *Bph15*-NIL following BPH infestation. Furthermore, our previous research has demonstrated the involvement of *OsEXO70E1* and *OsEXO70H3* in the *Bph6*-mediated resistance against planthoppers, as they facilitate exocytosis and enhance the strength of the cell wall (Guo et al., 2018; Wu et al., 2022). Notably, the analysis revealed a significant enrichment of the GO term “plant-type cell wall” in the R comparison (**Figure 5**). Moreover, the expression of *OsEXO70J1* was found to be downregulated in both the *wrky71* mutant and TN1 varieties, in contrast to *Bph15*-NIL, during the initial infestation of BPH (**Figure 7**). This finding suggests that *OsEXO70J1* may contribute to the resistance against BPH conferred by *OsWRKY71* in *Bph15*-NIL plants. Interestingly, the study found that the disease-resistance gene *RGA2* may also participate in BPH resistance conferred by *OsWRKY71* in *Bph15*-NIL plants. This implies that the plant immune system, traditionally recognized for its role in protecting against pathogens, may also be involved in plant-insect interactions. There may be shared downstream signaling mechanisms within the plant immune system that are relevant to these diverse types of interactions.

The SA and JA plant hormone signals are essential for defending rice against insects. However, the effectiveness of SA and JA in regulating rice defense against BPH is dependent on the specific genotype of the rice. The SA pathway is implicated in BPH resistance mediated by *Bph6*, *Bph9*, and *Bph14* (Du et al., 2009; Zhao et al., 2016; Guo et al., 2018). Moreover, the synergistic impact of SA and JA has been observed to enhance BPH resistance in *Bph6* plants, as reported by Guo et al. (2018). However, it has been discovered that *Bph15*-mediated BPH immunity is not influenced by the SA and JA pathways. This suggests the existence of additional mechanisms contributing to *Bph15*-mediated resistance. Additionally, *NPR1*, *PR1a*, *PR4*, *PR4b*, *PR10a*, and *RSOsPR10* are involved in a shared fundamental protective mechanism in both susceptible and resistant varieties of rice, except for *Bph15*-mediated BPH resistance.

Based on the findings of the study, it was observed that BPH insects that fed on the *wrky71* mutants exhibited a slight reduction in weight in comparison to those that fed on TN1 plants (**Figures 3C–E**). This implies that the *wrky71* mutants possess little resistance against BPH. Thus, it can be inferred that there exist other pathways implicated in *Bph15*-mediated BPH resistance signaling, and *OsWRKY71* does not exclusively regulate this resistance. The *Bph15*-NIL harbors two BPH resistance genes (*Bph3* and *Bph15*) (Lv et al., 2014; Xiao et al., 2016), thereby indicating the presence of multiple mechanisms contributing to BPH resistance. Further research should be undertaken to clone and characterize the resistance gene located in the recombination cold-spot region (Lv et al., 2014) to gain a more comprehensive understanding of their molecular mechanism against BPH. One potential approach involves the reconstruction of the genetic population capable of producing crossover in the recombination cold-spot region. This necessitates the identification of susceptible parents exhibiting a greater degree of chromosomal homology within the specified interval. Additionally, the deficiency in the *Bph15* physical map (Lv et al., 2014) could be addressed through the utilization of third-generation PacBio long-read sequencing to achieve completion.

In summary, the most significant finding to emerge from this study is that *OsWRKY71* plays a critical role in the *Bph15*-mediated BPH resistance in rice. This investigation significantly enhances our comprehension of the molecular mechanisms underlying *Bph15*-mediated BPH resistance. Further investigations should be conducted to successfully clone and identify all resistance genes in the *Bph15* locus.

## Data availability statement

The raw sequence files were deposited in the GSA database of the National Genomics Data Center (https://ngdc.cncb.ac.cn) with the accession number CRA011634 (https://ngdc.cncb.ac.cn/gsa/browse/CRA011634). 

## Author contributions

XL: Investigation, Writing – original draft, Writing – review & editing. JZ: Investigation, Writing – original draft, Writing – review & editing. XS: Investigation, Writing – review & editing. JY: Investigation, Writing – review & editing. LZ: Investigation, Writing – review & editing. JH: Investigation, Writing – review & editing. BD: Investigation, Writing – review & editing. WL: Funding acquisition, Investigation, Project administration, Resources, Supervision, Writing – original draft, Writing – review & editing.

## Supplementary material

Supplementary Figure 1 BPH-resistance scores of the *OsWRKY71* overexpression lines in the *Bph15*-NIL and Nipponbare backgrounds. **(A)** BPH-resistance scores of the *OsWRKY71* overexpression lines in the *Bph15*-NIL backgrounds. Bph15NIL-OE1, -OE2, and -OE3 are homozygous T_2_ progeny from independent transformants. **(B)** BPH-resistance scores of the *OsWRKY71* overexpression lines in the Nipponbare backgrounds. NIP-OE1, -OE2, and -OE3 are homozygous T_2_ progeny from independent transformants. Data represent the means ± SD from three separate experiments, each consisting of 15 seedlings per rice line. Significant differences (p < 0.05) are indicated by varying letters above the bars, as determined by a one-way ANOVA.

Supplementary Figure 2 Hierarchical cluster analysis of 1387 DEGs based on the log_2_(FC).

Supplementary Figure 3 GO enrichment analysis of 1387 DEGs.

Supplementary Figure 4 KEGG pathway enrichment analysis of 1387 DEGs.

Supplementary Figure 5 Representative KEGG pathway numbers of R0_R3, R0_R24, S0_S3, and S0_S24 comparisons.

Supplementary Figure 6 Volcano plots of R0_R3, R0_R24, S0_S3, and S0_S24 comparisons. Horizontal coordinate: log_2_(fold change), vertical coordinate: -log_10_(P-value). The representative DEGs were marked as boxes.
