# Supplementary figures and images for: Knockout of OsWRKY71 impairs Bph15-mediated resistance against brown planthopper in rice
Source: Front Plant Sci. 2023 Nov 2;14:1260526. doi: 10.3389/fpls.2023.1260526 (PMC10652391; doi:10.3389/fpls.2023.1260526)

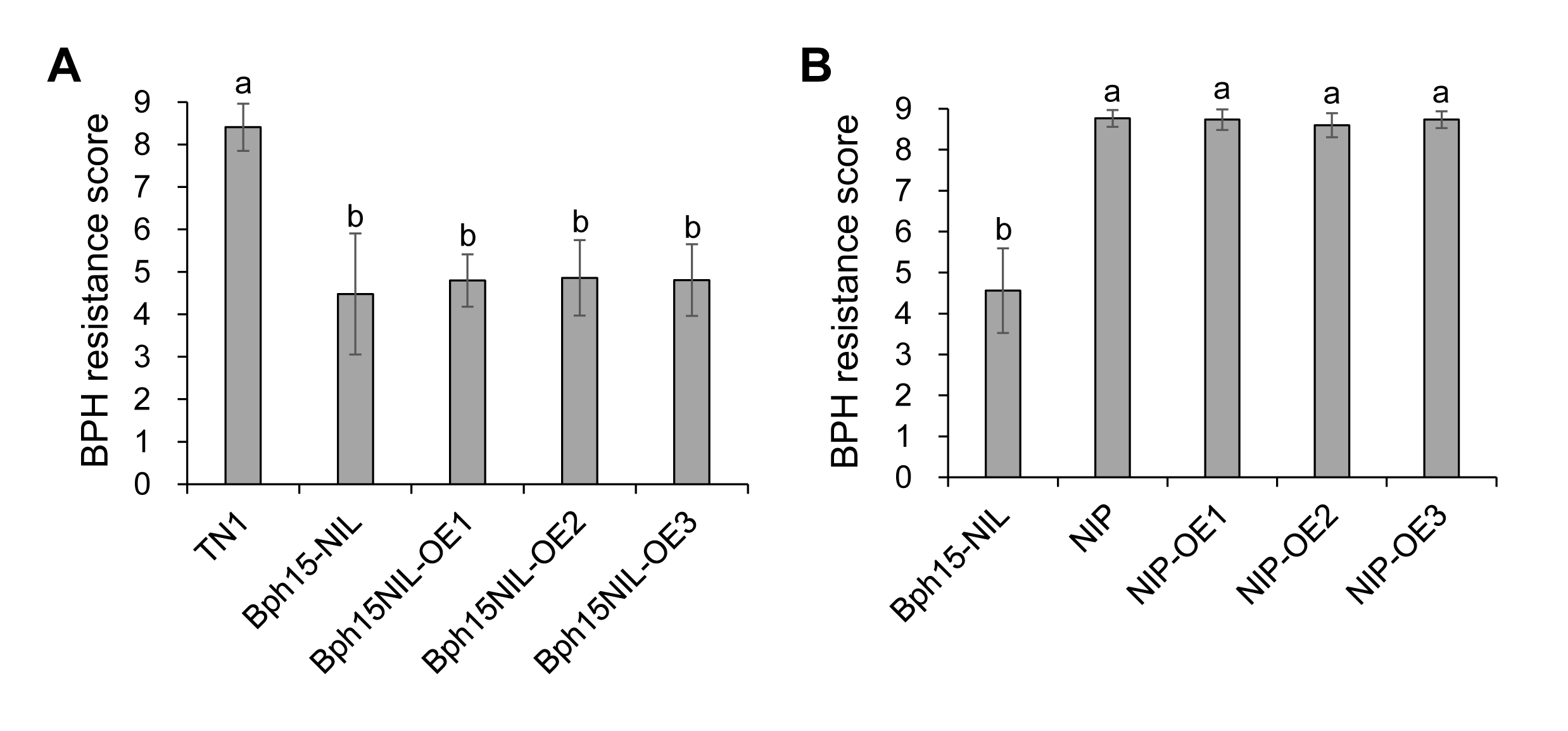

Supplement: Supplementary Figure 1 — BPH-resistance scores of the OsWRKY71 overexpression lines in the Bph15-NIL and Nipponbare backgrounds. (A) BPH-resistance scores of the OsWRKY71 overexpression lines in the Bph15-NIL backgrounds. Bph15NIL-OE1, -OE2, and -OE3 are homozygous T2 progeny from independent transformants. (B) BPH-resistance scores of the OsWRKY71 overexpression lines in the Nipponbare backgrounds. NIP-OE1, -OE2, and -OE3 are homozygous T2 progeny from independent transformants. Data represent the means ± SD from three separate experiments, each consisting of 15 seedlings per rice line. Significant differences (p < 0.05) are indicated by varying letters above the bars, as determined by a one-way ANOVA. [file Image_1.tif]

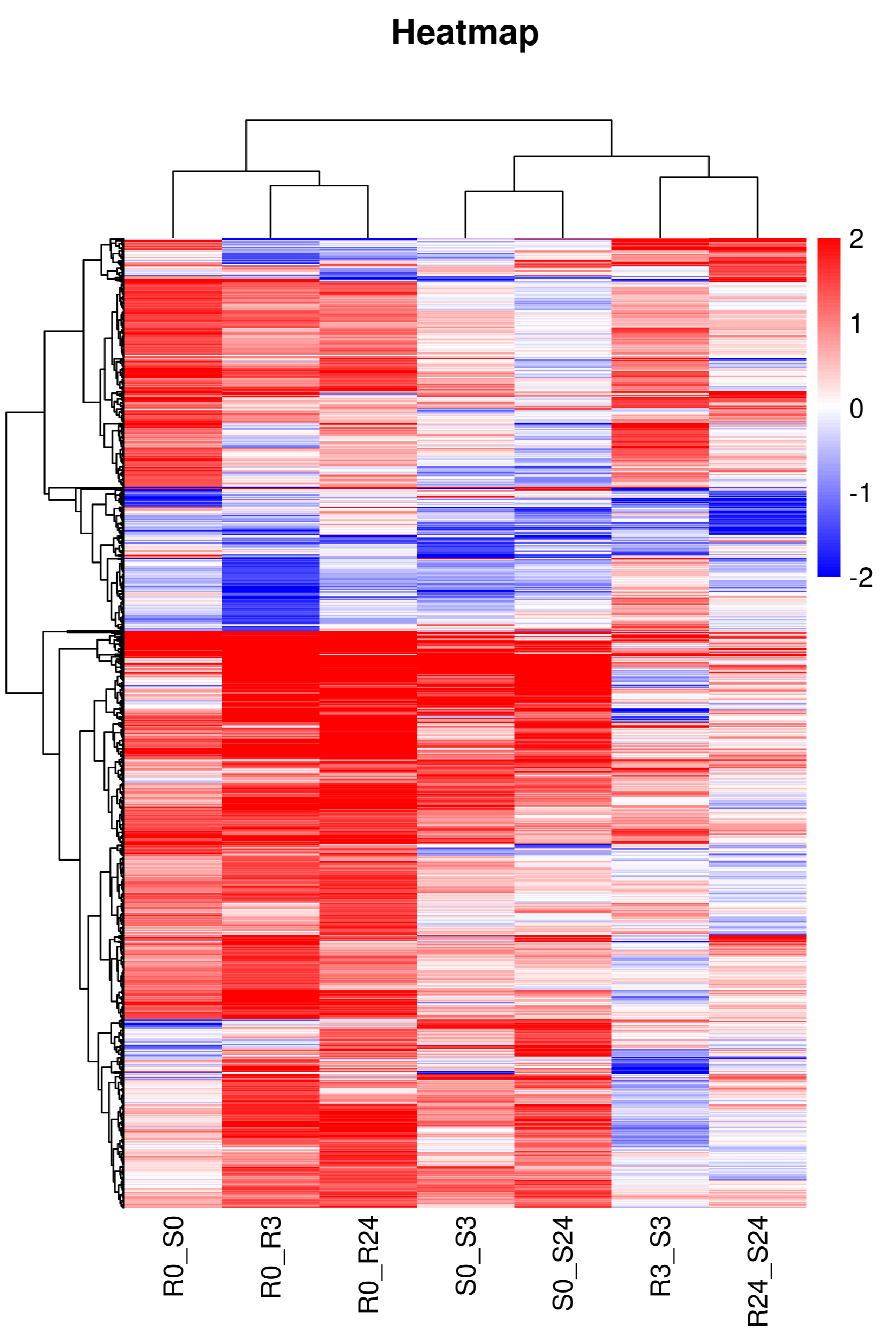

Supplement: Supplementary Figure 2 — Hierarchical cluster analysis of 1387 DEGs based on the log2(FC). [file Image_2.tif]

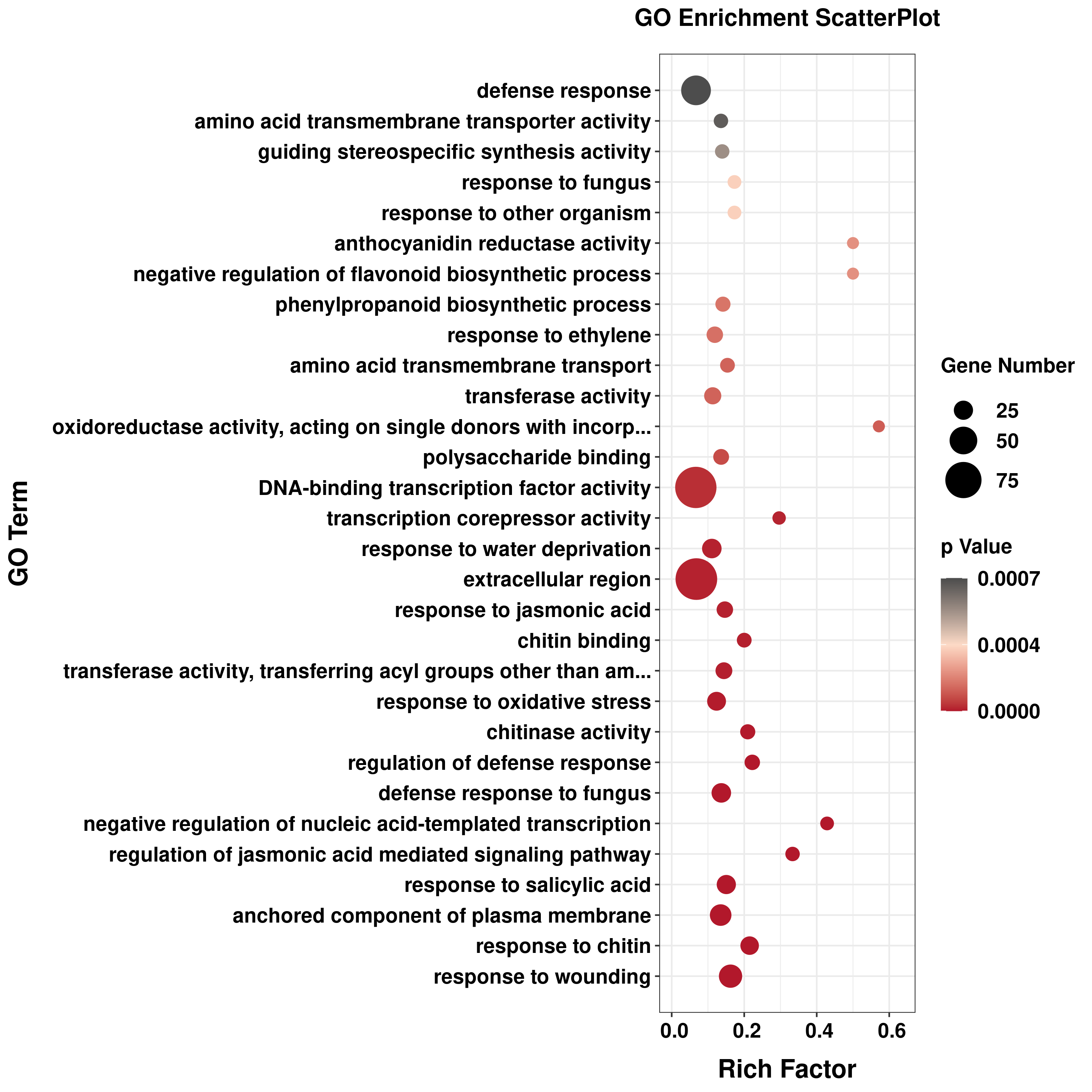

Supplement: Supplementary Figure 3 — GO enrichment analysis of 1387 DEGs. [file Image_3.tif]

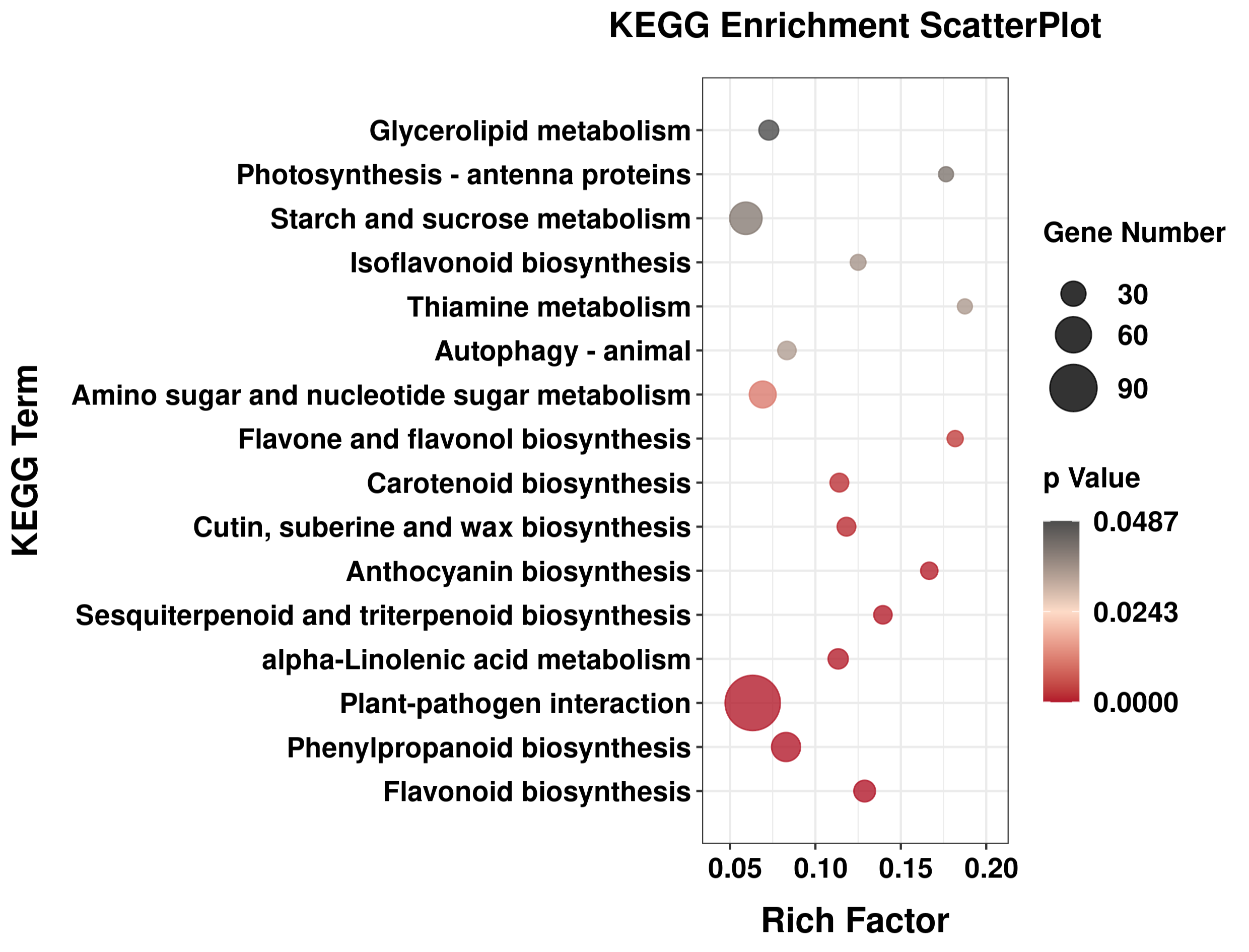

Supplement: Supplementary Figure 4 — KEGG pathway enrichment analysis of 1387 DEGs. [file Image_4.tif]

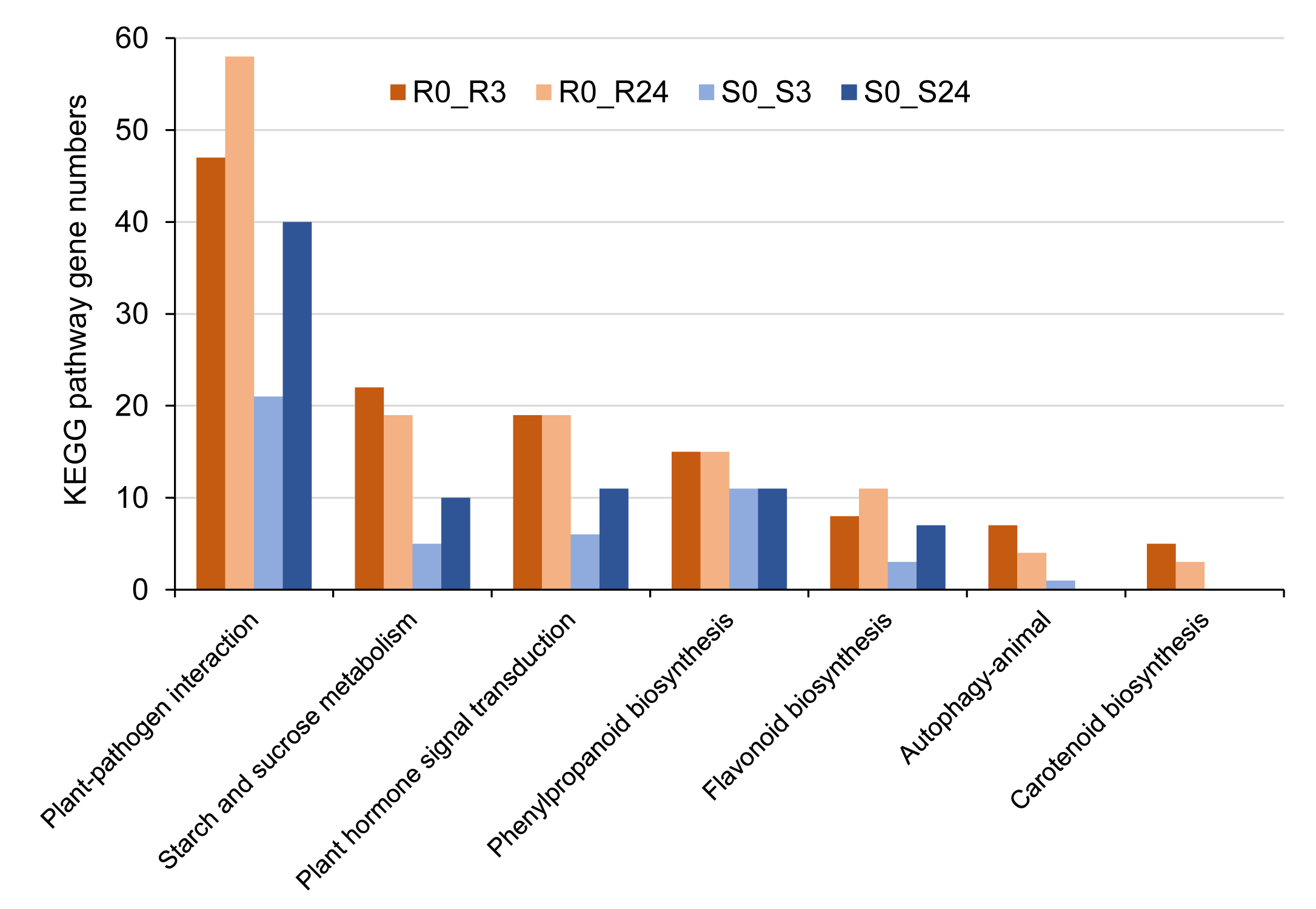

Supplement: Supplementary Figure 5 — Representative KEGG pathway numbers of R0_R3, R0_R24, S0_S3, and S0_S24 comparisons. [file Image_5.tif]

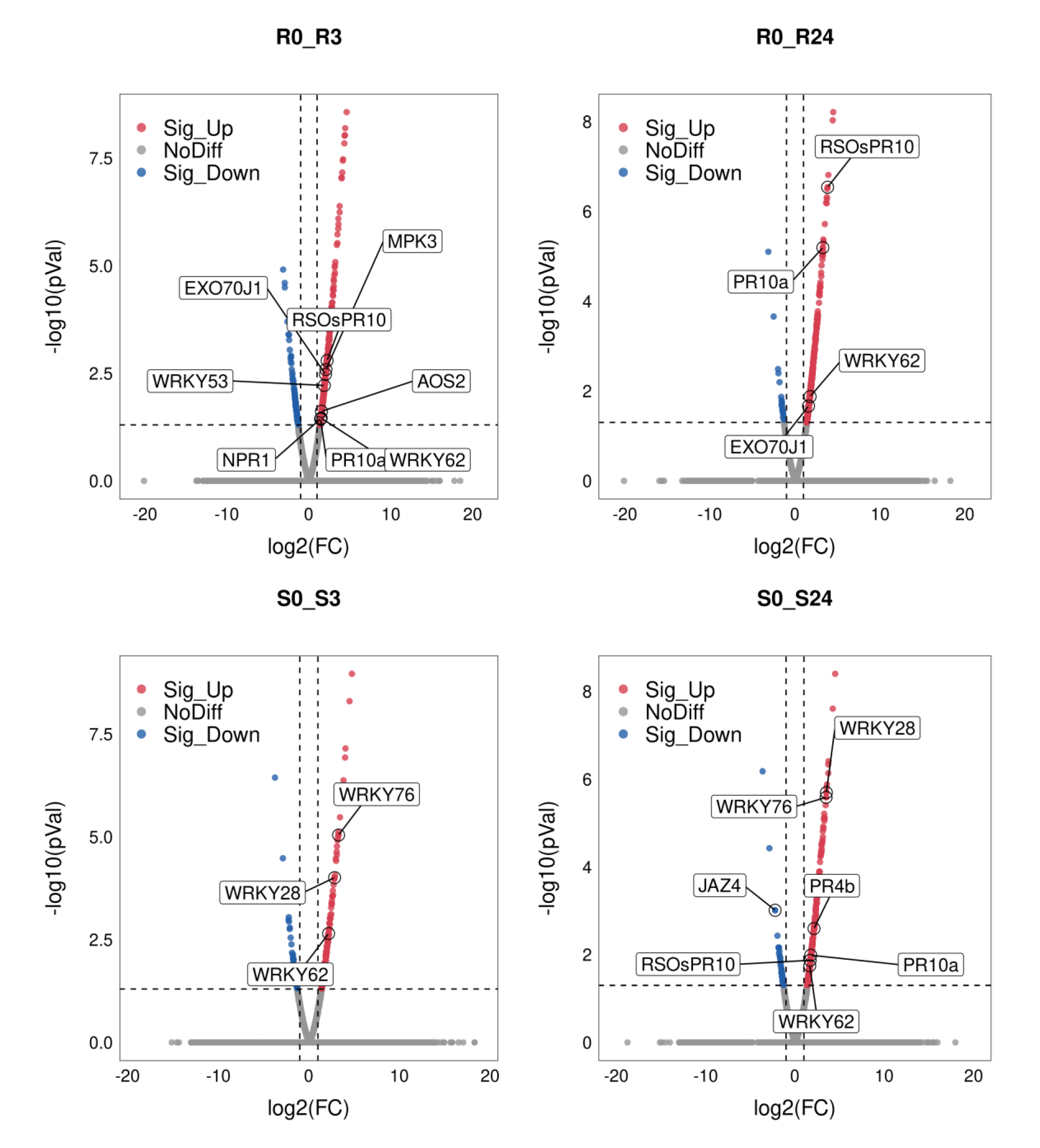

Supplement: Supplementary Figure 6 — Volcano plots of R0_R3, R0_R24, S0_S3, and S0_S24 comparisons. Horizontal coordinate: log2(fold change), vertical coordinate: -log10(P-value). The representative DEGs were marked as boxes. [file Image_6.tif]
